# Blasphemy laws and mental illness in Pakistan

**DOI:** 10.1192/pb.bp.112.042572

**Published:** 2014-02

**Authors:** Muzaffar Husain

**Affiliations:** 1 Kent Forensic Psychiatric Service, Dartford

## Abstract

There is emerging evidence that individuals who are mentally ill are overrepresented in the group of defendants prosecuted under the blasphemy laws of Pakistan. This article discusses the background of blasphemy legislation in Pakistan, and proposes causal interactions between underlying mental illness in the defendant and prosecution for blasphemy. It sketches possible legal safeguards for such blasphemy defendants with mental illness in mental health legislation.

Pakistan is a sprawling country in South Asia, approximately the size of France and the UK combined. With an estimated population exceeding 180 million people, it is the sixth most populous country in the world. The state was founded on 14 August 1947, following independence from Britain and partition from India. It is a federal state with five provinces of Sindh, Punjab, Baluchistan, Khyber Pukhtunkhwa and Gilgit-Baltistan, and the four Federally Administered Tribal Areas. In common with many other post-colonial nation states, Pakistan adapted its inherited British colonial legal and institutional framework in relation to its criminal laws. The Indian Penal Code passed by the British government for colonial India in 1860 became the Pakistan Penal Code^1^ after independence.

## Blasphemy laws

Historically, the Indian Penal Code 1860 was the first law in the Indian subcontinent that introduced the idea of ‘blasphemy’ in the criminal laws. For the first time, this law made it a criminal offence for anyone to defile religious symbols of any other religion.

The offences of blasphemy and blasphemous libel were fairly established as common law offences in the UK, at least from the 16th century, even though prosecutions were rare beyond the 19th century. The offences were abolished in England and Wales by the Criminal Justice and Immigration Act 2008, but they remain, to date, a part of the common law in Scotland and Northern Ireland. The remit of blasphemy in the UK was largely the Christian faith. However, in recent times, the main obstacle to prosecution has been the Human Rights Act 1998, which requires UK courts to interpret the criminal law in a manner compatible with the European Convention. In the case of blasphemy, it is argued that such an offence in common law imposes undue restrictions on individual rights to freedom of speech.

The legislative journey in Pakistan has been notably different. In the 1980s, there was a resurgence of state interest in the blasphemy laws, and there was an augmentation of the blasphemy laws, both in terms of expanding the remit of the laws to cover several further categories of behaviours that could be considered blasphemous, as well as an increase in the harshness of punishments on conviction.

Box 1 reflects some of the most frequently used sections of the blasphemy laws in the contemporary Pakistan Penal Code.^1^ Some of the offences, such as Section 295-C, are ostensibly offences of strict liability, i.e. they do not have *mens rea* requirements, and are not dissimilar to offences such as driving through a red traffic light, where what matters is the act itself, irrespective of intent. Sentences for blasphemy range from life imprisonment to death.

Section 295-C merits death as the only available sentence following a Federal Shariat Court ruling in 1994.^2^ In a similar ruling, the death penalty was added as a disposal to Section 295-B.

## Blasphemy defendants

There has been no systematic research on the numbers of blasphemy defendants in the Pakistani criminal justice system. This is as a result of, in part, the politically sensitive nature of all investigation of blasphemy cases in Pakistan, as well as the difficulty of capturing and verifying cases in the resource-poor setting of the penal estate. What research we have is conducted by individuals, working on their own, or in liaison with various human rights organisations, who take on advocacy roles for defendants on a case-by-case basis. Cases are also reported in the largely independent newsprint media. However, in the absence of any government-led investigation of these issues, there also has not been any external verification of this media reporting.

**Box 1** Pakistan Penal Code – some sections of the blasphemy laws

Section 295: Injuring or defiling place of worship, with intent to insult the religion of any class

Whoever destroys, damages or defiles any place of worship, or any object held sacred by any class of persons with the intention of thereby insulting the religion of any class of persons or with the knowledge that any class of persons is likely to consider such destruction, damage or defilement as an insult to their religion, shall be punished with imprisonment of either description for a term which may extend to two years, or with fine, or with both. (Introduced in 1860.)

Section 295-A: Deliberate and malicious acts intended to outrage religious feelings of any class by insulting its religion or religious beliefs

Whoever, with deliberate and malicious intention of outraging the religious feelings of any class of citizens of Pakistan, by words, either spoken or written, or by visible representations, insults the religion or the religious beliefs of that class, shall be punished with imprisonment of either description for a term which may extend to ten years, or with fine, or with both. (Introduced in 1927.)

Section 295-B: Defiling, etc., or Holy Qur'an

Whoever, wilfully defiles, damages or desecrates a copy of the Holy Qur'an or, of an extract therefrom, or uses it in any derogatory manner or for any unlawful purpose shall be punishable with imprisonment for life. (Introduced in 1982.)

Section 295-C: Use of derogatory remarks, etc., in respect of the Holy Prophet

Whoever by words, either spoken or written, or by visible representation or by any imputation, innuendo, or insinuation, directly or indirectly, defiles the sacred name of the Holy Prophet Muhammad (peace be upon him) shall be punished with death, imprisonment for life, and shall also be liable to fine. (Introduced in 1886.)

Nevertheless, even such limited sources of data indicate some interesting patterns.

There were 14 cases of blasphemy registered under the blasphemy laws between 1927 and 1984.^3^ But then between 1986 and 2010, the number of blasphemy defendants increased to 1274. This increase appears to have coincided with the widening of the blasphemy laws and an increase in the harshness of its punishments. A large proportion of these blasphemy defendants remain in prisons to date, with trials pending.

The same media sources also indicate that at least 51 blasphemy defendants have been murdered by the public following an allegation of blasphemy, some killed before a case could have been registered against them, some in police custody and others while they were serving a prison sentence. There has never been a government-led commission to investigate these reports.^3^

Sections 295-B and 295-C carry the death penalty, and have been reported to be the most frequently used sections of the blasphemy laws. Although death sentences have been awarded in several cases, there has not yet been a death penalty implemented in Pakistan.

## Political conflict and violence regarding the blasphemy laws

In contemporary Pakistan, the blasphemy laws remain a subject that polarises public opinion. There is a strong view that the laws are used maliciously to victimise minority communities, with the countervailing view that the self-proclaimed Islamic nature of the Pakistani state itself, requires, indeed mandates, such laws to justify its very existence as a state.

Senior politicians and judges who have advocated repealing of the blasphemy laws have been assassinated in recent times.^3^ Blasphemy defendants are held in secure settings within the prison estate because of the risks to their lives. Such real-world dangers also restrict the research activities of individuals interested in gathering systematic data on this issue. For example, on 4 January 2011 Mr Salman Taseer, the governor of Punjab province, was assassinated by his bodyguard for voicing a contrary opinion on the blasphemy law and supporting the female defendant of a blasphemy charge who had been sentenced to death. Later, his bodyguard, Mr Mumtaz Qadri, was convicted of the murder and sentenced to death, but he was also hailed as a hero for having performed his perceived religious duties to safeguard the faith, and there were public demonstrations in his support. Such was the intensity of feeling, that the judge who handed down the death sentence was reported in the press to have left the country, along with his family, after receiving death threats.^4^

## Mental illness and blasphemy offences – emerging evidence of a causal association

In this harsh sociopolitical setting, there has been an emerging acknowledgement that individuals who are mentally ill are also being disproportionately targeted under these laws, irrespective of their ethnicity or religious background. The evidence for this is derived from case reporting in the newspaper media. Again, unfortunately, there has been no external systematic examination of this issue.

On 3 July 2012, an unidentified man, widely reported by the police and the media to have been ‘mentally unstable’ was arrested for blasphemy in Bahawalpur district in Punjab province. A 2000 strong mob gathered outside the police station, and forcibly extracted him from police custody, doused him in petrol and set him on fire at the location where he was alleged to have burnt pages of the Quran. News reports using interviews from police officers described the man as confused, ‘laughing and chanting in custody’, and that he ‘had no idea what was going on’ even as the mob dragged him out of the police station to kill him.^5^

On 18 August 2012, a 14-year-old girl with Down syndrome was arrested from the outskirts of Islamabad because of allegations she had burnt pages of the Holy Quran.^6^ There was immediate panic in the area, but because of the media uproar following the disclosure of her age and mental impairments, she was later released on bail and given state protection. It turned out that a local religious leader had planted the evidence on her as part of a conspiracy to cause an exodus of her community members from the area. There appeared to be a clear association between her vulnerability as an individual with Down syndrome and her victimisation under the blasphemy law.

On 21 December 2012, a 35-year-old unidentified man was arrested by the police in the Dadu district of Sindh province.^7^ He was charged under Section 295-B of the blasphemy laws. According to the station police officers, a complaint was lodged with them that stated that the suspect, a stranger in the area, allegedly entered a local mosque at 03.00 h, ‘threw away a rosary’ and ‘torched the Quran’. When the mosque watchman made attempts to stop him, the suspect allegedly ‘threatened to kill’ him and his children. He was placed in police custody but was unable to reveal any personal details to the police for over 5 h. In the meanwhile, a 1000 strong mob gathered, and proceeded to attack the police station. They extracted the suspect from custody, and, in a violent fury, beat him to death. The descriptions of his behaviour at the time of the alleged offence, and later, when he was in police custody, suggest the presence of mental illness.

## Clinical formulations of mental illness and blasphemy

The entire spectrum of psychopathology lends itself to various behavioural infringements that could fall foul of the blasphemy laws in place in Pakistan. Individuals with psychotic disorders, such as mania and schizophrenia, can present symptoms of grandiose and bizarre delusional systems of being of divine origin, behavioural disinhibition and lack of insight, which place them at risk of prosecution under these laws. Individuals with autism, with varying degrees of intellectual disability, are another diagnostic group also at risk of not being able to follow social rules of due reverence and regard for what the community holds sacrosanct. Neurotic disorders such as obsessive-compulsive disorder can also create overbearing and compelling pressures on individuals to blaspheme in some instances, and this can have serious legal consequences if the targets of the blasphemy are revered by the community.

The association of mental illness and blasphemy allegations can also be more complex. Sexual abuse of vulnerable individuals with intellectual disabilities has on occasion been masked by allegations of blasphemy by abusers when they fear discovery. The lack of any legal protections for such vulnerable blasphemy defendants ensures that their testimony is extracted when they fear for their lives, and the sex abuse issue is concealed behind the allegations of blasphemy. In other cases, physical abuse can be an interacting factor, where the blasphemy allegations are raised against an individual with autism for example, when various attempts to physically discipline the individual have failed, and have, instead, resulted in severe physical injuries, which could lead to criminal prosecution against the abusers, unless blasphemy is raised as the reason why the discipline was implemented in the first place.

The most commonly cited criticism of the blasphemy laws is that they are used by the majority community to victimise religious minorities. Nevertheless, it is often the case that the person who is alleged to have committed the blasphemy has underlying vulnerabilities that explain why that person in particular stands so charged, and not others within the same disadvantaged community. The basis of this vulnerability can often be some form of mental illness. It is a separate matter whether this illness could benefit from treatment, but what is clear is that such individuals require some form of legal and social protections by virtue of their mental illness.

## Current mental health defences in Pakistan criminal courts

The association between mental illness and blasphemy has not been entirely unacknowledged in the criminal courts of Pakistan. If the cases progress to the higher courts, the defendant's underlying mental illness is likely to be raised as a relevant factor in the case.

The Pakistan Penal Code contains the mental health defence of legal insanity, which has been successfully raised in some cases.

Section 84 of the Pakistan Penal Code states that ‘Nothing is an offence which is done by a person who, at the time of doing it, by reason of unsoundness of mind, is incapable of knowing the nature of the act, or that he is doing what is either wrong or contrary to law’. It can be raised by the defence and must be proved before the judge on a balance of probabilities. And indeed in the case of *Shahbaz Masih v. State*, heard in the Lahore High Court,^8^ the blasphemy defendant was seen by eye witnesses to have trampled pages of the Holy Quran, and throwing the pages on graves. He was handed over to the police with the torn pages of the Quran in his possession. He was charged under Section 295-B of the blasphemy laws. Medical evidence was obtained for him, which supported the view that he was of ‘unsound mind’ at the time of the act, and that he had a history of mental illness. He was accordingly acquitted, and released by the court.

Even so, given the generally poor psychiatric services available in the country and the dearth of training in the provision of expert psychiatric evidence in courts, such cases are rare. It must be noted, however, that even as some blasphemy offences are framed so as to resemble offences of strict liability, once legal insanity has been proved the courts can take a humanitarian stance and grant an absolute discharge of the defendant.

## Proposed safeguards for blasphemy defendants with mental illness in mental health legislation

The simplest and most obvious manner to remedy such human rights violations, and the stigma and abuse of individuals with mental illness who happen to fall foul of these laws, is to advocate for a repeal of the blasphemy laws from the statute books in *to to*. Such advocacy has been pursued for several decades, with few results. However, as individuals with mental illness are now being specifically targeted under these laws, some sections of the Pakistani mental health community have recently taken the initiative to galvanise broad-level support for the introduction of specific safeguards for blasphemy defendants who are mentally ill in the mental health laws of the country, as a legal protection issue for vulnerable individuals with mental illness.

Upon independence, Pakistan inherited the (British India) Lunacy Act of 1912 as its mental health legislation. This archaic law remained the fundamental mental health law of the land until 2001 when the Pakistan Mental Health Ordinance was passed. Since then, the project for the introduction of meaningful mental health legislation has progressed in fits and starts. Recent amendments in the constitution of Pakistan have caused a new wave of mental health legislation in every province. This has presented a new opportunity for concerned mental health professionals to introduce special protections for vulnerable individuals with mental illness in the criminal justice system.

One such example is in the province of Sindh. The Sindh Mental Health Bill of 2012 was drafted on the initiative of concerned mental health professionals and the assistance and advice of various mental health experts, lawyers, human rights activists and government functionaries in the country, and a few mental health law experts in the UK.^9^ Building on the principle of diversion of defendants who are mentally ill out of the criminal justice system into healthcare settings, it included a clause for the mandatory psychiatric assessment of all blasphemy defendants.^10^

The Sindh Mental Health Ordinance 2013 was passed by the interim provincial government on 8 May 2013. As expected, the government balked at the introduction of any law which could be perceived as being directly lenient for blasphemy defendants, even on mental health grounds. Section 53(3) of the Sindh Mental Health Ordinance 2013 obliquely refers to issue (Box 2). It must be noted that the blasphemy laws of Pakistan are coded under Chapter XV of the Pakistan Penal Code 1860, and Section 53(3) of the Sindh Mental Health Ordinance 2013 refers to Chapter XIV of the Pakistan Penal Code instead, which refers to essentially non-religious moral and public nuisance offences. Modest though its scope appears, it could still be construed as the first ever introduction of any legal protections for such mentally ill defandants.

**Box 2** Relevant clauses of legislation

*Sindh Mental Health Ordinance 2013*^11^

Section 53(3): Where any person is detained under provisions of Chapter XIV of the Pakistan Penal Code 1860, a Board or any two members of any such Board shall visit him in order to ascertain his state of mind and shall make a report as to the state of mind of such person to the competent court.

*Pakistan Penal Code 1860*^1^

Chapter XIV: Of offences affecting the public health, safety, convenience, decency and morals.

At the same time, even such a proposed strategy is far from secure in accomplishing the aim of guaranteeing safeguards for such blasphemy defendants with mental illness. Mental health is often far from being at the forefront of the service delivery agenda of the provincial government. There is an historical apathy towards mental healthcare that must also be surmounted alongside other issues specific to blasphemy and mental illness. It is also argued that the incorporation of safeguards for blasphemy defendants in mental health law runs the risk of attracting the ire of pro-blasphemy violent forces against patients with mental illness.

There are also questions raised in relation to the limited resources in the country committed to psychiatric care. Who will assess these defendants in the criminal justice system? And where will they be diverted for treatment, if this is what is required? And will this alternative arrangement be safe enough for them and for the staff who look after them?

It is difficult to foretell all the consequences of introducing legal protections for individuals with mental illness in society. The history of legally safeguarding the interests of people with mental illness in the several mental health laws of the UK since 1775 has arguably been no different in this respect. Yet legal protections for vulnerable individuals who are mentally ill in every society must begin somewhere. The solution for this in the case of blasphemy defendants with mental illness in Pakistan will have to be discovered within the Pakistani context.

Nevertheless, it remains the universal humane position that if it is in fact an illness that has caused the person to behave ‘blasphemously’ on this occasion, then no matter how terrible the offence might be perceived to be by others, it follows that the person ought to be given some basic legal and social protections and, where possible, appropriate treatment for their condition, instead of punishment.
